# Launching a CDMO in Brazil aiming to develop biopharmaceuticals for clinical trials

**DOI:** 10.1590/1678-9199-JVATITD-2022-0017

**Published:** 2022-06-06

**Authors:** Rui Seabra Ferreira, Marcelo Marcos Morales, Pasqual Barretti, Benedito Barraviera

**Affiliations:** 1 Center for the Study of Venoms and Venomous Animals (CEVAP), São Paulo State University (UNESP), Botucatu, SP, Brazil.; 2 Department of Tropical Diseases and Diagnostic Imaging, Botucatu Medical School (FMB), São Paulo State University (UNESP), Botucatu, SP, Brazil.; 3 Carlos Chagas Filho Institute of Biophysics, Federal University of Rio de Janeiro (UFRJ), Rio de Janeiro, RJ, Brazil.; 4 Secretariat of Research and Scientific Training, Ministry of Science, Technology, and Innovation (MCTI), Brasília, DF, Brazil.; 5 Department of Internal Medicine, Botucatu Medical School (FMB), São Paulo State University (UNESP), Botucatu, SP, Brazil.; 6 Rector, São Paulo State University (UNESP), São Paulo, SP, Brazil.

**Keywords:** Contract development and manufacturing organizations (CDMO), Translational research, Clinical trials, Good manufacturing practices (GMP), Biopharmaceuticals

## Abstract

The innovation timeline is expensive, risky, competitive, time-consuming, and labor-intensive. In order to overcome such challenges and optimize financial resources, pharmaceutical companies nowadays hire contract development and manufacturing organizations (CDMO) to help them. Based on the experience acquired first from the development of two biopharmaceuticals, the Heterologous Fibrin Sealant and the Apilic Antivenom, and more recently, during their respective clinical trials; the Center for the Study of Venoms and Venomous Animals (CEVAP) proposed to the Ministry of Health the creation of the first Brazilian CDMO. This groundbreaking venture will assist in converting a candidate molecule - from its discovery, proof of concept, product development, up to pilot batch production - into a product. The CDMO impact and legacy will be immense, offering service provision to the public and private sector by producing validated samples for clinical trials and academic training on translational research for those seeking a position in pharmaceutical industries and manufacturing platforms.

There is a wide gap between a scientific discovery and its practical application in the drug development process. In the pharmaceutical industry, technological advances have been obtained based on scientific innovation and the persistent work of inventors and industrialists. This has always been the starting point. Nowadays, in addition to such process, science must also predict the means by which the industrial application can be most fully achieved. The scientific literature worldwide has been discussing this topic since the late 1940s [1].

Translational clinical research refers to the work focused on transforming research into practical actions, ensuring that new clinical technologies actually reach the patients they are intended for and that these actions and knowledge are implemented correctly. The production of a new drug, a common endpoint for “bench to bedside” translational research, is just the starting point for this area of research [2].

The innovation schedule is expensive, risky, competitive, time-consuming, and labor-intensive. It takes between 15 and 20 years to develop a product, in addition to a huge amount of human and financial resources. For a candidate molecule to become a drug available to the population, in general, one billion dollars are invested.

As drugs become more complex, pharmaceutical companies face new challenges for development and manufacturing. Faster innovation and production are required to bring solutions to the market, keep investments under control, and reduce costs, which are priority actions for most companies [3].

To overcome such challenges and optimize financial resources, nowadays big pharma companies hire contract development and manufacturing organizations (CDMO) to help them. These companies were formed to work together with large pharmaceutical companies and research and development (R&D) groups to create products, as well as to validate and test new candidate molecules. Therefore, pharmaceutical industries eliminate the necessity of investing in additional infrastructure for this purpose. CDMO are the ones that handle the outsourced manufacturing of drug substances. In addition, they carry out all the innovative and developmental work required to the manufacturing of a product. Consequently, development, production and analysis remain part of CDMO function in the process, whereas the industries are able to bring down the costs relative to maintaining facilities and specialized personnel for this.

In Brazil, the main generation of wealth is the export of commodities, mainly those produced in agriculture. In 2021, agribusiness generated a trade superavit of more than 60 billion dollars. On the other hand, health - intensified by the Sars-Cov-2 pandemic - had an estimated deficit of 30 billion dollars in 2020. This situation was mainly due to Brazilian necessity to import basic hospital supplies (masks, gloves, respirators), diagnostic kits, medicines, and vaccines [4, 5].

Governments have observed this grim situation for decades. In order to change it, in 2011, guidelines were established for the so-called “Plano Brasil Maior” - its motto was “innovate to compete, compete to grow” - which included the health industrial-economic complex as one of the strategic priorities for the national development and wealth generation [6].

To start the long process of behavior changes in the 2010s, the Brazilian government selected projects focusing on human health with translational potential for a medium-term resource plan.

São Paulo State University (UNESP - https://youtu.be/3sBXavLi578) was one of the protagonists of this story. Thus, in 2010, a project was submitted to the Ministry of Health of Brazil and the phase I/II study “Treatment of chronic venous ulcers with Fibrin Sealant derived from snake venom” was approved (CNPq, proc. n. 563582/2010-3) in the amount of BRL 3,764,638.28. UNESP's counterpart would be the construction of a pilot laboratory (worth BRL 2,031,875.00), in compliance with good manufacturing practices (GMP), to continue the following phases of the research. In 2013, another projected was submitted, the phase I/II of *"*Treatment of envenoming by massive Africanized honeybees (*Apis mellifera*) stings with the new Apilic Antivenom”, to the Ministry of Health of Brazil and it was approved (CNPq, proc. n. 401170/2013-6) raising funds of BRL 786,200.00.

Both National Council for Scientific and Technological Development (CNPq) funding calls aimed to select proposals to support projects on clinical research on new drugs, products, or strategic resources for the Unified Health System (SUS). Moreover, select proposals should contribute to scientific, technological and innovative development of Brazil. Both products were developed from the bench to the bedside within UNESP, employing national and low-cost technology [7-10]. The first study was completed in January 2016 (https://youtu.be/y2cvGH7X6D8) and the second one in November 2018 (https://youtu.be/y6ho6M0amA8). The results were published in high-impact open-access journals in 2020 [11-12], and in 2021 [13]. The analysis showed that the products were safe and with promising efficacy. A phase III multicenter clinical trial is required to validate the findings, to request registration with ANVISA, and to distribute the products throughout Brazil via SUS network. Figures 1 and 2 show respectively the application of the Heterologous Fibrin Sealant in chronic venous ulcers and the Apilic Antivenom packaging.

**Figure 1.**
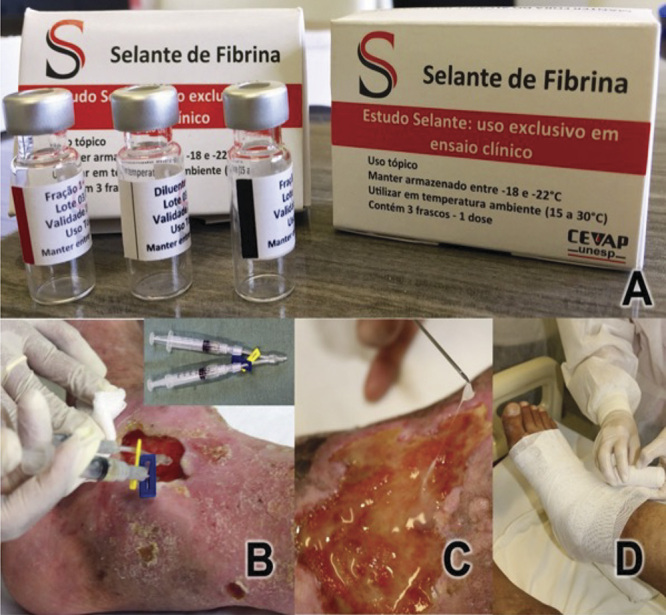
Application of the Heterologous Fibrin Sealant in chronic venous ulcer. Reprinted from "Treatment of chronic venous ulcers with Heterologous Fibrin Sealant: a phase I/II clinical trial" by Abbade et al. [12].

**Figure 2.**
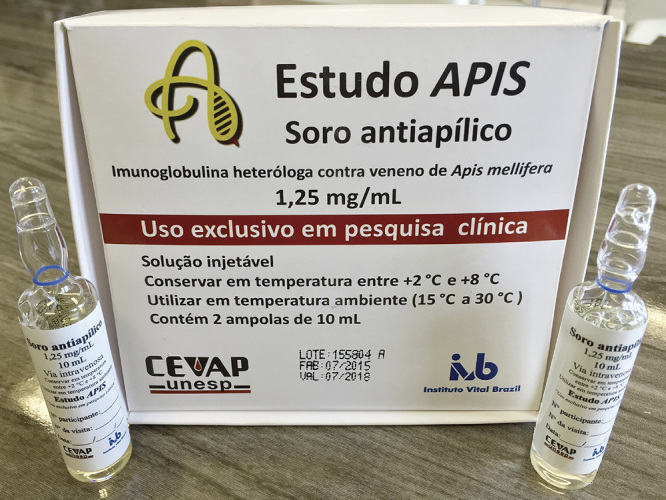
Apilic Antivenom packaging. Reprinted from "Single-arm, multicenter phase I/II clinical trial for the treatment of envenomings by massive Africanized honey bee stings using the unique Apilic Antivenom" by Barbosa et al. [13].

The development of innovative and competitive products must be anchored in consolidated and reproducible science. This is a great challenge for scientists: to produce reproducible and quality science. Baker [14] in 2016 challenged 1,576 world-renowned researchers to reproduce their own experiments and those of other researchers. According to Baker, more than 70% of the researchers have tried and failed to reproduce another scientist's experiments, and more than half have failed to reproduce their own experiments. Baker [14] also asked: how much-published work in your field is reproducible? The predicted reproducibility of the surveys was on average as follows: 50% for medicine, 70% for biology and chemistry, and 80% for physics and engineering. In 2018 Barba [15] defined the terms repeatability, replicability, and reproducibility. They are respectively: repeatability (experiment carried out by the same team and with the same experimental configuration); replicability (experiment carried out by different teams and with the same experimental setup), and reproducibility (experiment carried out by different teams with different experimental setup). Considering this heated discussion, in 2019 the National Academies of Science, Engineering, and Medicine [16] published an extensive guideline consensus to explain the main differences between reproducibility and replicability.

All this knowledge is necessary when one wants to develop “translational science” in order to cross the “valley of death”. The development of any “candidate molecule” has the following timeline: discovery, basic research, applied research, product development, and production. To reach this happy ending, it is necessary to cross the “valley of death” that lies between basic and applied research [2, 17-19]. 

The Center for the Study of Venoms and Venomous Animals (CEVAP) of UNESP (https://youtu.be/goaBkbnTqCg) dared to cross this barrier by developing two successful products over decades, the Heterologous Fibrin Sealant and the Apilic Antivenom. These two phase I/II clinical trials were approved by Anvisa to be carried out without the need for good manufacturing practices (GMP). For this, it was necessary to prepare complete reports of the products called “investigator's brochure”, to standardize the supply chain of active pharmaceutical ingredients and to carry out toxicological and stability tests [20-22]. On that occasion, it was determined by Anvisa that for future phase III clinical trials the samples should be produced in a GMP environment.

As there was no such institution in Brazil capable of developing samples for clinical research, in April 2017 the Secretariat of Science and Technology and Strategic Inputs of the Ministry of Health of Brazil (SCTIE/MS) approved the construction of the Factory for the Production of Pilot Batches for Clinical Research at UNESP Campus de Botucatu. The construction began on February 7, 2021 (Additional files 1-4). This manufacturing plant, within a Brazilian public university, will act as a CDMO, not only helping pharmaceutical industries to meet deadlines or market demand but also to save time and reduce infrastructure costs. Accordingly, it must have validated equipment, adequate facilities, including a clean area, and professionals with specialized skills. The CDMO will have to prepare pilot batches for clinical trials, submit reports to validate new drugs to regulatory agencies and, finally, prepare projects to scale finished products without additional labor expenses. The impact and legacy of this work for UNESP and for Brazil will be immense and divided between the provision of services to the public and private sectors - producing validated samples for clinical trials - and academic training to professionals to work on manufacturing plants of the pharmaceutical industry (https://www.youtube.com/watch?v=tJIXFS05vxo). The full operation of this initiative should occur within the next two years.

## Supplementary material

The following online material is available for this article:

Additional file 1. Approval in April 2017 of the project of the CDMO by DECIIS-SCTIE/MS in Brasília, DF, Brazil.

Additional file 2. Signing of the contract for the construction of the CDMO in January 2022 at CEVAP/UNESP in Botucatu, SP, Brazil.

Additional file 3. Front 3D image of the CDMO of CEVAP/UNESP.

Additional file 4. Lateral 3D image of the CDMO of CEVAP/UNESP.
