# Supplementary material for: Launching a CDMO in Brazil aiming to develop biopharmaceuticals for clinical trials
Source: J Venom Anim Toxins Incl Trop Dis. 2022 Jun 6;28:e20220017. doi: 10.1590/1678-9199-JVATITD-2022-0017 (PMC9171928; doi:10.1590/1678-9199-JVATITD-2022-0017)
Supplement: Additional file 1. [file 1678-9199-jvatitd-28-e20220017-s1.pdf]

J Venom Anim Toxins incl Trop Dis

https://doi.org/10.1590/1678-9199-JVATITD-2022-0017

Supplementary Material to "Launching a CDMO in Brazil aiming to develop biopharmaceuticals for clinical trials"

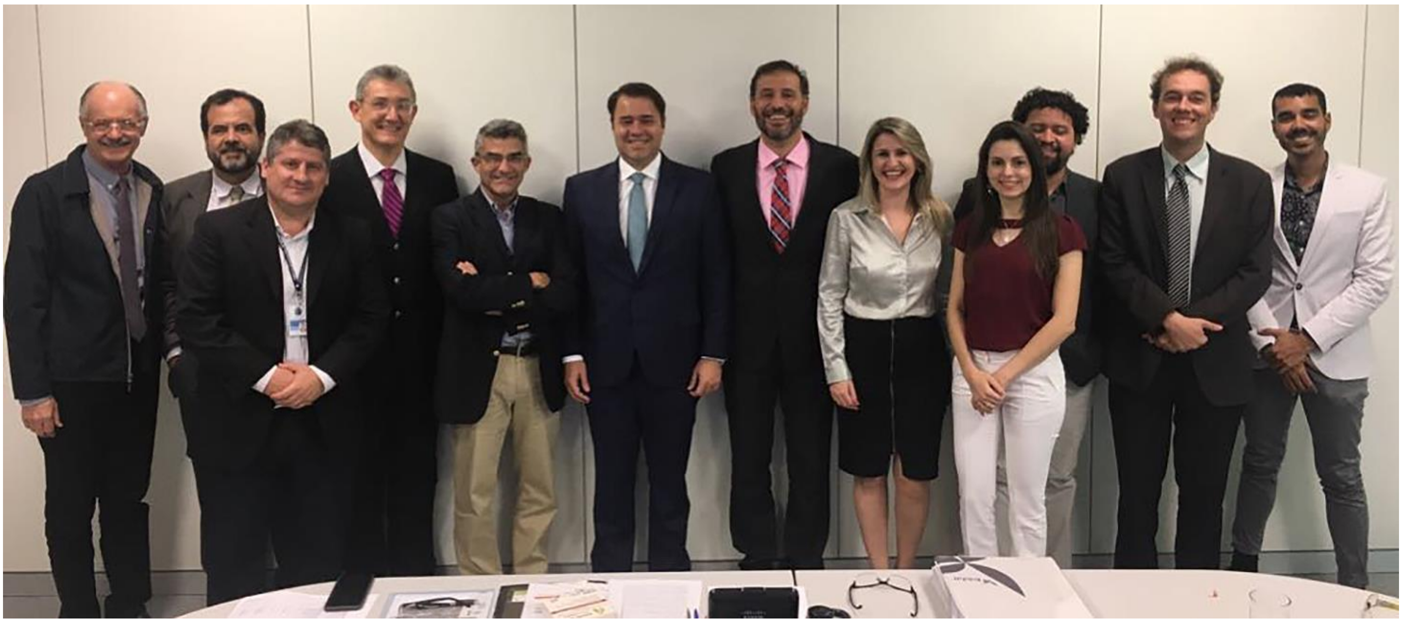

Additional file 1. Approval in April 2017 of the project of the CDMO by DECIIS-SCTIE/MS in Brasília, DF, Brazil.
